# MiR-218 regulates epithelial–mesenchymal transition and angiogenesis in colorectal cancer via targeting CTGF

**DOI:** 10.1186/s12935-018-0575-2

**Published:** 2018-06-08

**Authors:** Weijian Lun, Xiongjian Wu, Qiliang Deng, Fachao Zhi

**Affiliations:** Guangdong Provincial Key Laboratory of Gastroenterology, Inst. of Gastroenterology of Guangdong Province, Department of Gastroenterology, Nanfang Hospital, Southern Medical University, Guangzhou, 510515 China

**Keywords:** Colorectal cancer, miR-218, CTGF, EMT, Angiogenesis

## Abstract

**Background:**

Endothelial-to-mesenchymal transition (EMT) and angiogenesis play important roles in colorectal cancer (CRC) development. Connective tissue growth factor (CTGF) has been reported to promote several kinds of cancer progression and miR-218 has been identified as a tumor suppressor miRNA. However, little is known about the function of miR-218 in CRC. Here we investigated the effects of miR-218 on EMT and angiogenesis process in CRC cells. As well, the relation between miR-218 and CTGF was identified. The mechanism of miR-218’s function was illustrated.

**Methods:**

CRC cell lines were transfected with miR-218 mimics. Proliferation, migration and angiogenesis were identified by MTT assay, Transwell assay, colony formation assay and tube formation assay. Protein and mRNA expression levels of associated genes were measured by Western blotting and RT-PCR. Dual luciferase assay was used to determine the relation of miR-218 and CTGF.

**Results:**

miR-218 was down-regulated in CRC cell lines and over expression of miR-218 could significantly inhibit EMT and angiogenesis. CTGF was a direct target of miR-218. Up regulation of CTGF level after miR-218 transfection could sufficiently rescue the suppression effects on EMT and angiogenesis.

**Conclusion:**

miR-218 directly targets CTGF and inhibits its expression, leading to suppression on EMT and angiogenesis of CRC cells. miR-218 might be used as potential therapeutic strategy for CRC treatment.

## Background

Colorectal cancer (CRC) is the most common type of cancers in the current world and 90% of its mortality is accounted by metastasis [27]. Primary CRC originates from epithelial cells and during its progression, cancer cells are believed to obtain a mesenchymal phenotype that promote them to migrate from the primary tumor site to distant organs [29]. The process is known as the epithelial-to-mesenchymal transition (EMT). Besides this, angiogenesis also plays a key role in tumor growth and progression [9]. Increased angiogenesis not only supplies tumor cells with nutrition, but also provides routes for cancer cells metastasis [8].

Connective tissue growth factor (CTGF) is a member of the CCN family, secreted multifunctional proteins that contain high levels of cysteine [21]. CTCF has been well studied for its involvement in tissue remodeling in various diseases, including cancer [24]. Previous reports have identified CTGF as a fibrogenic cytokine that is up-regulated in wound healing and fibrotic lesions [23]. In kinds of cancers, the pleiotropic functions of CTGF have been demonstrated [10]. CTGF over-expression has been proven to be associated with poor prognosis in several kinds of tumors, including B-cell acute lymphoblastic leukemia [25], gastric cancer [20], glioma [35], prostate cancer [36], and pancreatic cancer [3]. In breast cancer, studies have shown that CTGF cooperates with other genes to promote metastasis, and high level of CTGF correlated with advanced tumor stages [34]. In osteosarcoma, CTGF has been proved to facilitate angiogenesis by regulating miR-543/angiopoietin 2 signaling [37].

MicroRNAs (miRNAs) are small, non-coding RNAs that are broadly conserved among species. miRNAs function primarily to regulate gene expression at post-transcriptional level through specifically binding to the 3′-untranslated region (3′UTR) of their mRNAs [2]. Ectopic expression of miRNAs has been identified in many types of cancers [5]. miR-218 belongs to the SLIT gene family and commonly acts as a tumor suppressor gene [13]. Reports have demonstrated that miR-218 was significantly down-regulated in cancer samples from patients and played an inhibitive role in cancer development [32]. In colorectal cancer, miR-218 has been shown to target metastasis related gene MACC1 and inhibit cancer progression [14]. However, it is still not fully understood about miR-218 function on EMT and angiogenesis of colorectal cancer.

In the present study, we demonstrated that miR-218 was down-regulated in CRC cell lines and tissues. Over-expression of miR-218 was able to inhibit EMT process, leading to a repression of cell proliferation, migration, and invasion. Moreover, our results indicated that miR-218 could regulate cancer cell drive angiogenesis in vitro. Further study discovered CTGF was a direct target of miR-218. Consequently, our findings provide new insights into the mechanism of miR-218 in CRC development and potential application of miR-218 as a therapeutic target for CRC.

## Materials and methods

### Cell culture

The human CRC cell lines SW620, SW480, HCT8 and HCT116 and human normal colon epithelial cell line NCM460 were obtained from the American Type Culture Collection (ATCC; USA). The umbilical vein endothelial cell line HUVEC and 293T cell line were from Cell Bank of Type Culture Collection of Chinese Academy of Sciences (Shanghai, China). Cells were cultured in Dulbecco’s Eagle’s Medium (DMEM, with high glucose and l-glutamine; Gibco, USA) supplemented with 10% Fetal Bovine Serum (FBS; Invitrogen, USA) and 1% Penicillin/Streptomycin (P/S; Thermo Fisher, USA) and incubated at 37 °C in 5% CO_2_. Recombinant Human CTGF Protein was purchased from R&D systems (9190-CC-050, USA) and added to culture medium at a final concentration of 10 ng/mL for 30 min before indicated experiments.

#### Patient samples

CRC tumor samples and adjacent normal tissues were collected from patients in Nanfang Hospital, China and used under approved protocol from the ethics committees in Southern Medical University. Written informed consent was obtained from all subjects. CRC tumor stages were divided according to TNM staging system.

### Real-time PCR

Total RNA was extracted from cultured cells with TRIzol reagent (Tiangen, China) according to the manufacturer’s instruction. cDNA was synthesized with the M-MLV reverse transcriptase (Invitrogen, USA). Real-time PCR was performed in a Real-Time Thermocycler7500 (Applied Biosystems, USA) with a SYBR Green Real-Time PCR Kit (Tiangen, China). For normalization, U6 and GAPDH were used as the endogenous controls for miR-218 and indicated genes, respectively. Fold changes were determined by 2 − ΔΔCt method. The sequences for real-time PCR were: forward 5′ CACCCACCCACATACATAC 3′, reverse, 5′ CATCTCCTCCTCTTCCCT 3′ (VEGFA); forward5′ CTTATGAGCGAGAATGGG 3′, reverse, 5′ TAGGTTGTTGGGTTGTTT 3′ (ANGPT2); forward 5′ ACGGATTTGGTCGTATTG 3′, reverse, 5′ GGAAGATGGTGATGGGATT 3′ (CTGF); forward 5′ AACGGATTTGGTCGTATTG 3′, reverse 5′ GGAAGATGGTGATGGGATT 3′ (GAPDH). The relative expression levels of miRNA and mRNAs in each sample were tested in triplicated.

### MTT assay

Growth rates of cells were measured by 3-(4, 5-dimethylthiazol-2-yl)-2, 5-diphenyl tetrazolium bromide (MTT) assay. Cells (3 × 10^3^/well) were seeded in 96-well culture plates and incubated overnight. After being washed, 0.5 mg/ml of MTT was added at 0, 24, 48 and 72 h, respectively. 4 h later, the culture medium was removed and dimethyl sulfoxide (DMSO) was added to solubilize the crystals. The absorbance was measured at a wavelength of 490 nm using a microplate auto reader (BioTek Instruments, USA). Independent experiments were repeated in triplicate.

### Transwell assay

For the Transwell assay, 1.0 × 10^5^ cells were placed in the top chamber of Transwell plate (BD Biosciences, USA). The cells were seeded in serum-free media and 10% FBS was added to the culture medium in the lower chamber. After 24 h of culturing, the cells remained in the upper layer were removed and those had migrated through the membrane were stained with a dye solution of 20% methanol and 0.1% crystal violet. The cells were then imaged under a light microscope (Olympus) and ten individual fields were counted per insert. The results were presented as an average of three separate experiments.

### Colony formation assay

Colony formation assay was used to analyze the ability of anchorage-independent cell proliferation. The bottom layer contained 0.4% w/v agarose in DMEM with 10% FBS and was added into culture dish. The top layer contained 8 × 10^3^ cells, 0.2% w/v agarose in DMEM with 10% FBS and was added onto the solidified bottom layer. Cells were seeded as single cell into the soft agar and cultured for 14 days. Colonies were visualized by light microscope (Olympus). The assay was repeated three independent times.

### Tube formation assay

HUVECs (1 × 10^5^/well) were seeded into Matrigel-coated wells in a 24-well plate. Supernatants from indicated groups and fresh medium (1:2) were mixed and added as conditioned medium (CM) 8 h later, photographs were taken. Only perfectly continuous tubes between two branching points were considered as a tube. The assay was repeated three independent times.

### Western blotting assay

Cells were washed three times using cold PBS and lysed in RIPA buffer with protease inhibitors. Approximate 0.03 mg of protein was separated with 10% SDS-PAGE gel and blotted into nitrocellulose membranes. Then membranes were blocked with 5% nonfat dried milk blocking buffer at room temperature for 1 h and incubated with diluted primary antibodies (1:1000) against GAPDH (#5174, Cell Signaling Technology, 1:1000), E-cadherin (ab15148, Abcam, 1:1000), α-catenin (ab51032, Abcam, 1:1000), Vimentin (#5741, Cell Signaling Technology, 1:1000), Fibronectin (ab2413, Abcam, 1:1000), VEGFA (ab51745, Abcam, 1:1000) and ANGPT2 (ab65835, Abcam, 1:1000) at 4 °C overnight. Then membranes were washed by TBST 3 times and incubated with horseradish peroxidase-conjugated secondary antibodies (1:5000) at room temperature for 1 h. Protein bands were visualized by a Molecular Imager ChemiDoc XRS System (Bio-Rad Laboratories, USA) α-catenin.

### Dual luciferase assay

The 3′UTR of the CTGF mRNA containing either the wild type or mutated miR-218 binding site was cloned into the restriction sites of PGL3 luciferase reporter vector (Promega, USA). A total of 8 × 10^4^ 293T cells were seeded into 24-well plates and co-transfected with reporter plasmid and indicated miRNA mimics. Luciferase assay was carried out using the Dual Luciferase Assay Kit (Promega, USA) following the manufacturer’s instructions. Three wells of cells were used for each group.

### Statistical analysis

GraphPad Prism version 6.0 software (GraphPad, USA) was used to analyze differences between two groups with student’s t-test. A two-tailed P < 0.05 was significant, and data were presented as the mean ± SD.

## Results

### miR-218 is down-regulated in CRC cell lines

**Fig. 1:**
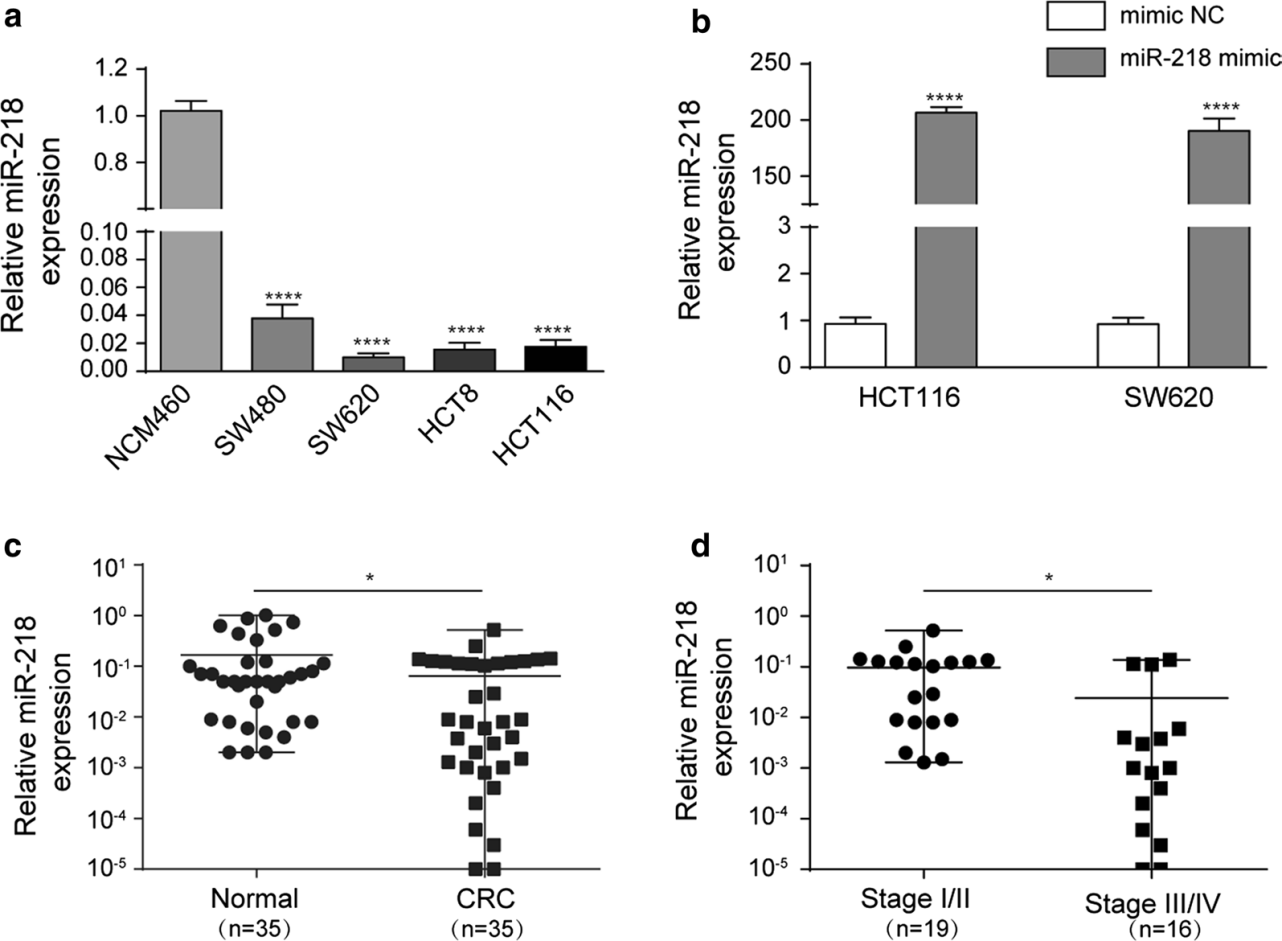
miR-218 was down-regulated in CRC cell lines and tissue. **a** Compared to human normal colon epithelial cell line NCM460, miR-218 expression was significantly decreased in CRC cell lines, including SW620, SW480, HCT8, and HCT116. **b** Transfection with miR-218 mimics significantly increased its level in SW620 and HCT 116, compared to control groups N = 3. **c** Levels of miR-218 were obviously decreased in CRC patients’ samples, compared to adjacent normal tissues. N = 35. **d** In the late stage CRC tumors (N = 16), miR-218 was dramatically decreased compared to early stage CRC tumors (N = 19). *P < 0.05, ***P < 0.0001, t-test

To determine whether miR-218 is dysregulated in CRC cell lines, we evaluated its expression level by miRNA RT-PCR experiments. The result showed that compared to normal colon epithelial cell line NCM460, the levels of miR-218 in several CRC cell lines, including SW480, SW620, HCT8, and HCT116 were remarkably lower (Fig. 1a). SW620 and HCT116 were chosen for the further studies. To determine the function of miR-218 in CRC cell lines, we transfected SW620 and HCT116 with miR-218 mimic or mimic NC respectively. The efficiency was detected by RT-PCR and the result indicated that the levels of miR-218 were significantly increased after transfected with miR-218 mimic compared with negative control (mimic NC) (Fig. 1b). Furthermore, in 35 cases of CRC tumor samples, miR-218 was down-regulated compared to respective adjacent normal tissues (Fig. 1c). Meanwhile, miR-218’s level was altered in tumor samples from different stages. In early stages of I and II, miR-218 expression was relatively higher than that in late stages of III and IV (Fig. 1d).

### miR-218 inhibits CRC proliferation and migration in vitro

**Fig. 2:**
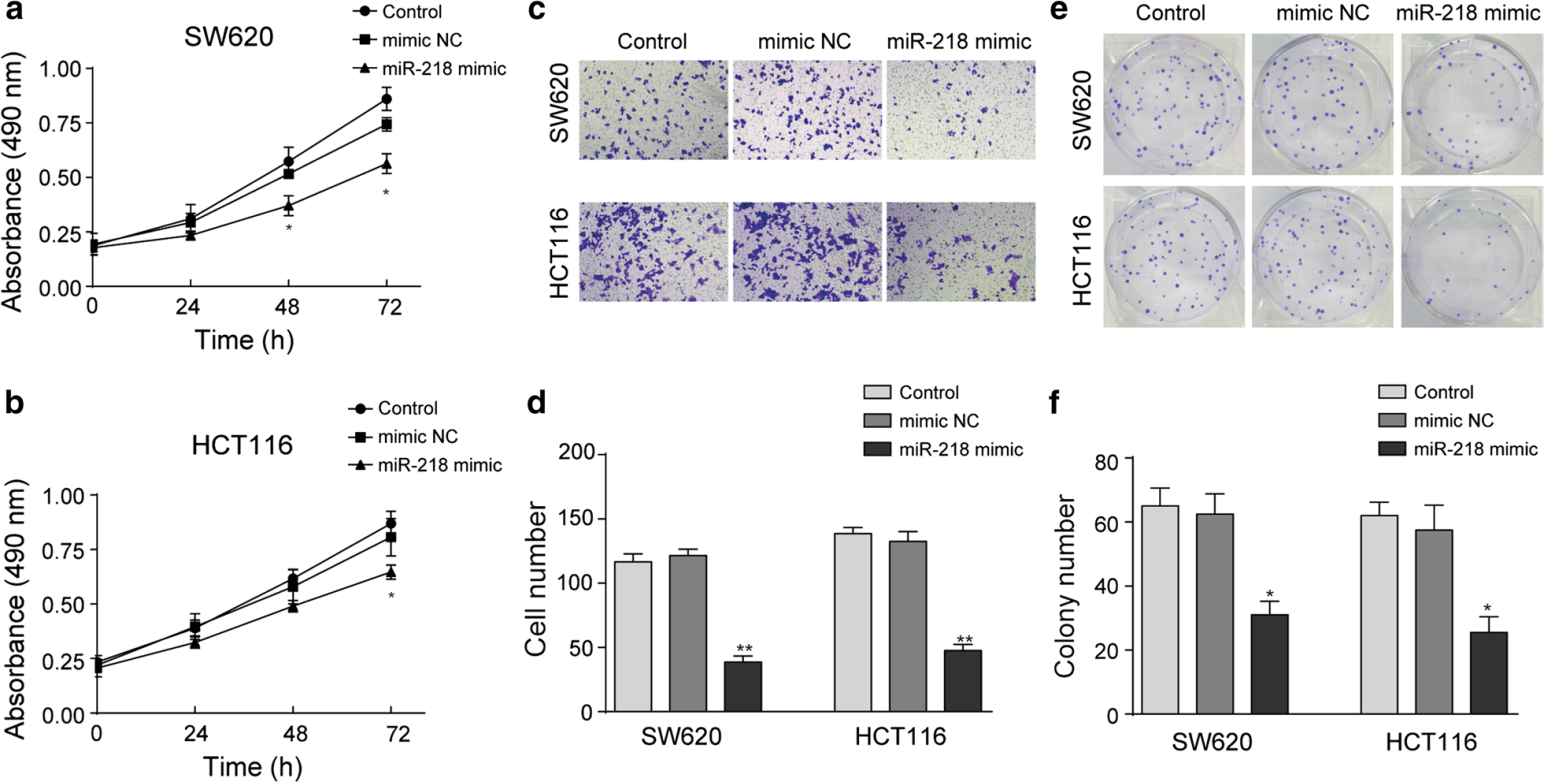
miR-218 suppressed migration of CRC cells. **a**, **b** When transfected with miR-218 mimic, the growth rate of SW620 (**a**) and HCT116 (**b**) was remarkably inhibited compared to control groups. **c**, **d** Over expression of miR-218 could obviously suppress the ability of CRC cells migration by Transwell assay. **e**, **f** Up regulation of miR-218 sufficiently decreased the amount of colonies formed by CRC cells. N = 3, *P < 0.05, **P < 0.01, t-test

Cancer progression includes a cohort of cellular processes, including cell proliferation, migration, invasion and tumor nude formation. To assess effect of miR-218 on CRC proliferation and migration, a series of experiments were conducted. As shown in Fig. 2a, b, over expression of miR-218 significantly inhibited proliferation rates of SW620 (48 and 72 h) and HCT116 (72 h) in different time points, compared to mimic NC groups. Meanwhile, in Transwell assay, both SW620 and HCT116 exhibited strong migration ability, while transfected with miR-218 mimic, the amount of migrated cells were dramatically deceased, as shown in Fig. 2c, d. Similar results were achieved in colony formation assay. With elevated miR-218 level, the numbers of colony formed by SW620 or HCT116 were significantly decreased, compared to mimic NC groups (Fig. 2e, f). Taken together, these results revealed that miR-218 significantly inhibits CRC proliferation and migration in vitro.

### miR-218 suppresses tumor angiogenesis in vitro

**Fig. 3:**
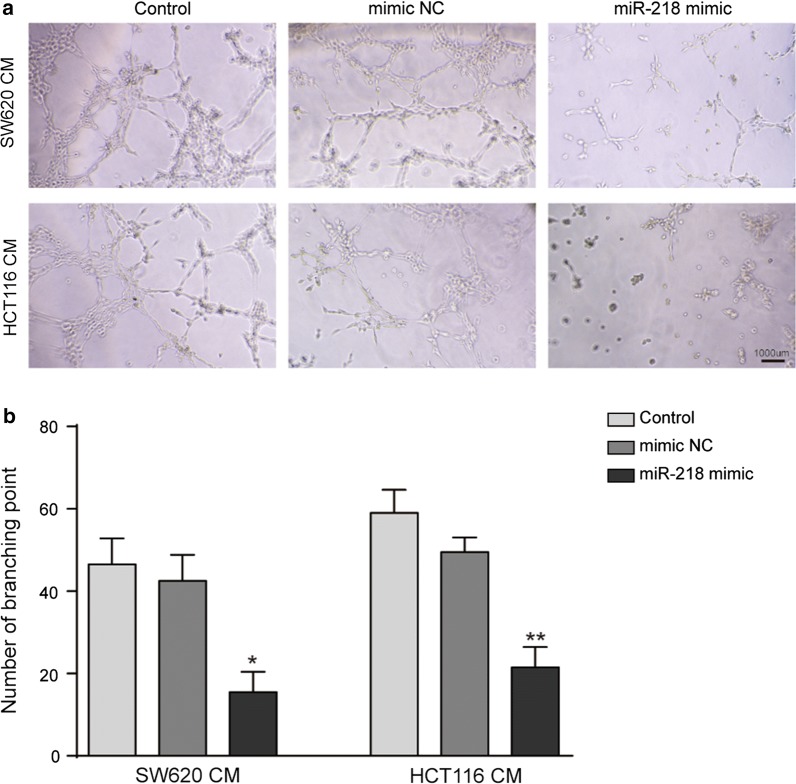
miR-218 inhibited tube formation of HUVECs. **a**, **b** HUVECs were treated with supernatants from indicated group. The tubes number was significantly decreased when treated with that from miR-218 over expression CRC cells. N = 3, *P < 0.05, **P < 0.01, t-test

Angiogenesis also plays important role in tumor development. To investigate whether miR-218 could regulate tumor angiogenesis, the in vitro tube formation assay was employed, which can measure the capacity of endothelial cells to form capillary-like structures. Human umbilical vein endothelial cells (HUVECs) were treated with conditioned medium (CM) from SW620 or HCT116 transfected with miR-218 mimic or controls. As shown in Fig. 3a, b, miR-218 over-expression significantly suppressed tube formation ability of HUVECs by decreasing the number of branching point, indicating an inhibitive effect on tumor angiogenesis of CRC.

### miR-218 regulates proteins associated with EMT and angiogenesis

**Fig. 4:**
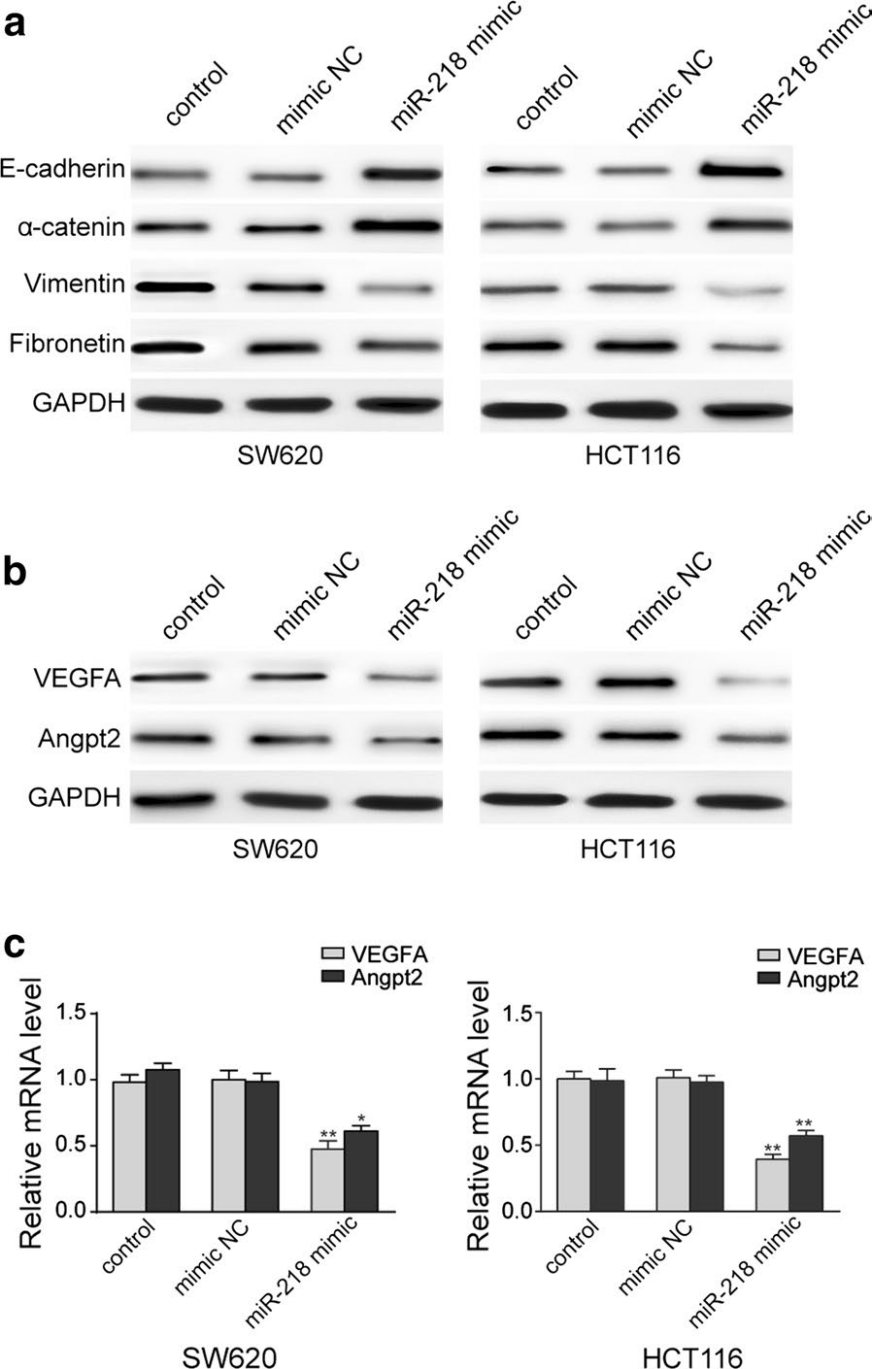
miR-218 regulated the expression of EMT and angiogenesis associated genes. **a** Over expression of miR-218 could increase the level of E-cadherin and α-catenin, while decrease the expression of Vimentin and Fibronectin in SW620 and HCT116. **b**, **c** Up regulation of miR-218 significantly inhibited the protein (**b**) and mRNA (**c**) levels of VEGFA and ANGPT2 in SW620 and HCT116. N = 3, *P < 0.05, **P < 0.01, t-test

To further identify the mechanism of miR-218 on CRC progression, we detected the protein levels of proteins associated with EMT and angiogenesis. As shown in Fig. 4a, for both SW620 and HCT116 cells, over expression of miR-218 remarkably increased the level of E-cadherin and α-catenin, which are typical proteins expressed by epithelial cells, while decreased the level of Vimentin and Fibronectin, which are typical proteins expressed in mesenchymal cells, compared to control groups. As for angiogenesis process, miR-218 overexpression significantly suppressed the protein levels of VEGFA and ANGPT2 in SW620 and HCT116, which play important role in tumor angiogenesis (Fig. 4b). The same trends were identified for their mRNA level by RT-PCR (Fig. 4c). These results demonstrated that miR-218 suppressed CRC progression via regulating genes associated with EMT and angiogenesis.

### miR-218 directly targets CTGF to inhibit CRC development

**Fig. 5:**
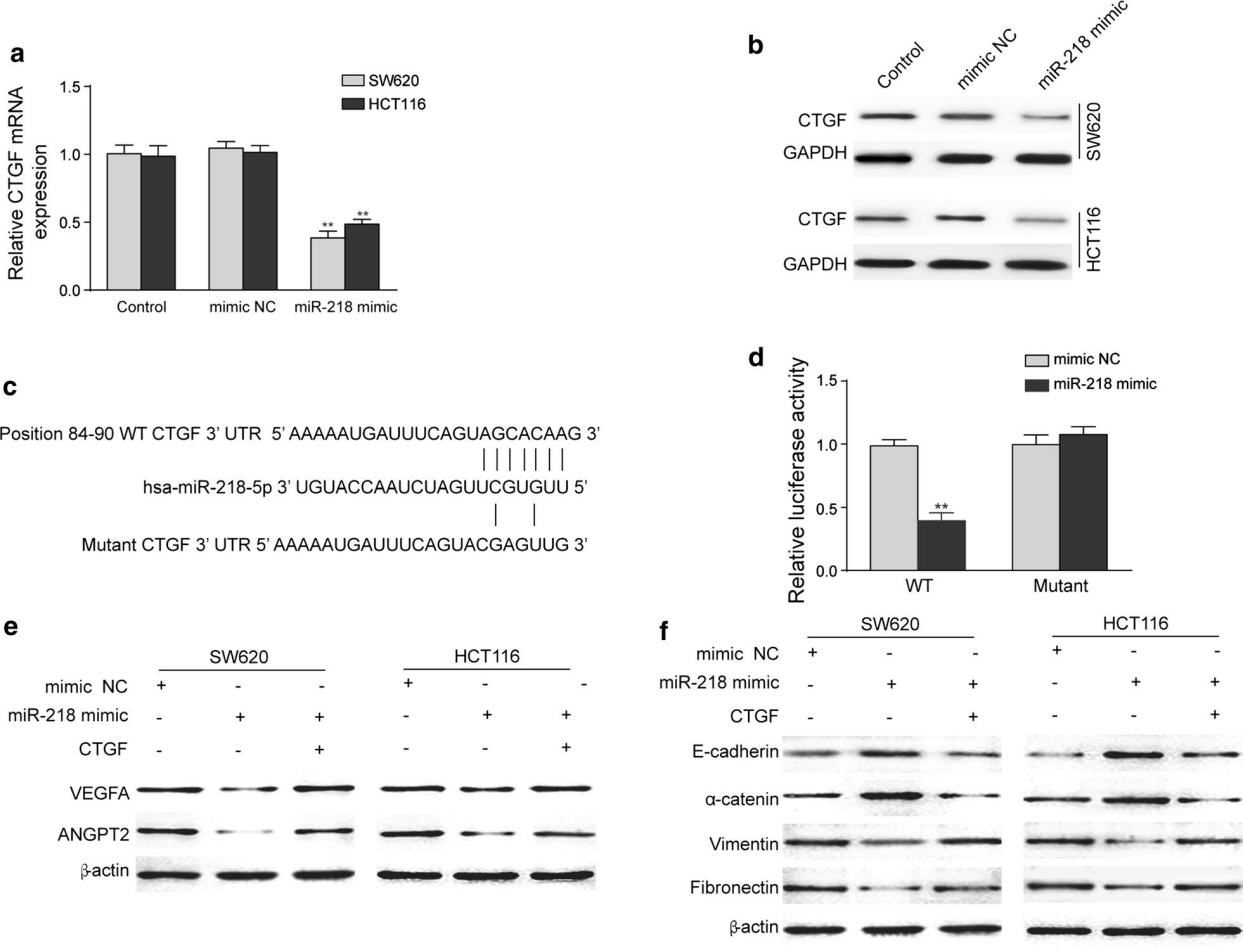
CTGF was a direct target of miR-218 and could restore its inhibition effects on CRC progression. **a**, **b** miR-218 was able to suppress the mRNA (**a**) and protein (**b**) expression of CTGF. **c**, **d** miR-218 could inhibit the luciferase reported activity to 50%, demonstrating that it directly targeted CTGF. **e** CTGF treatment rescued the inhibition effects on EMT associated proteins brought by miR-218 over expression. **f** CTGF treatment restored the inhibition effects on VEGFA and ANGPT2 brought by miR-218 over expression. N = 3, **P < 0.01, t-test

As CTGF plays a key role in tumor development and it is predicted to be targeted by miR-218 by Targetscan database, we further validated if miR-218 directly regulates CTGF expression. Western blot and RT-PCR results demonstrated that overexpression of miR-218 was able to inhibit protein and mRNA levels of CTGF in both SW620 and HCT116 cell lines (Fig. 5a, b). Wild type or mutant 3′UTR of CTGF containing miR-218 binding site were cloned into the reporter system (Fig. 5c). As shown in Fig. 5d, miR-218 significantly suppressed luciferase activity of wild type CTGF 3′UTR by 50%, while no inhibitive effect was observed for mutant one. These results proved that CTGF was a direct target of miR-218 and its level was inhibited in CRC cell lines when miR-218 was over expressed.

To further illustrate miR-218 exert inhibitive effect via attenuating CTGF in CRC, recombinant CTGF was supplied into culture medium in SW620 and HCT116. Our results showed that CTGF supplement rescued the inhibited EMT and angiogenesis induced by miR-218 over-expression, as demonstrated by the decrease of E-cadherin and α-catenin protein levels and increase of Vimentin, Fibronectin, VEGFA and ANGPT2 protein levels (Fig. 5e and f). These results further supported that miR-218 suppress CRC progression via directly inhibiting CTGF expression and the downstream pathways.

## Discussion

miRNAs are small non-coding RNAs, which are usually 18–22 nucleotides. They were first discovered in nematodes in 1990s [19]. To date, more than 1000 miRNAs have been identified in human [4]. miRNAs have been well studied for their important roles in the regulation of gene expression by targeting mRNAs for translational repression or degradation and demonstrated to be involved in many cancer biological processes, including tumor initiation, metastasis and angiogenesis. Many miRNAs have been proved to act as tumor suppressors and the decreased level of these miRNAs results into promotion of tumor development. For example, miR-23b inhibits cancer cell metastasis by targeting MAPK pathway [39] and miR-190 suppresses tumor angiogenesis by directly targeting VEGF [12].

Previous studies have demonstrated that miR-218 functions as a tumor inhibitor in different kinds of human cancers. In gastric cancer, miR-218 is down-regulated and able to inhibit tumor metastasis by targeting the Robo1 receptor [31]. The progression of nasopharyngeal cancer and cervical cancer are negatively correlated with miR-218 level [1, 17]. In addition, miR-218 is significantly decreased in prostate cancer tissues from patients and could inhibit cancer cells growth and promote apoptosis [22]. Meanwhile, in colorectal cancer, miR-218 is proved to target pro-tumorigenesis gene MACC1 [14] and BMI-1 [13] to inhibit CRC development.

EMT plays a crucial role in many stages in tumor progression [16]. Cancer cells undergoing EMT are endowed with more aggressive phenotypes, such as mesenchymal and stem cell like features. This transition results in the acquisition of malignant properties, such as migration and invasion [38]. Many genes and pathways have been implicated in inducing EMT in cancer cells. Meanwhile, these pathways are usually active in other processes as well, including cell proliferation, apoptosis. EMT is characterized by a decrease in the expression of proteins that promote cell–cell contact, such as E-cadherin and α-catenin, as well as an increase in the level of mesenchymal markers such as Vimentin and Fibronectin. It has been demonstrated that EMT constitutes an early stage of metastasis [30], in which cancer cells detach from the primary tumor and invade through the basement membrane into the circulation. In our study, we disclosed the role of miR-218 and mechanism of suppressing EMT in CRC. Consistent with its function in other kinds of cancers, miR-218 was down-regulated in CRC cell lines and could repress EMT process in CRC cells. Over-expression of miR-218 was demonstrated to inhibit CRC cell proliferation, migration and colony formation ability. As for the EMT markers, E-cadherin and α-catenin were increased after transfected with miR-218 mimic, while expression of Vimentin and Fibronectin were significantly decreased.

In addition to EMT process, angiogenesis is also important for tumor progression. The formation of neovascular is a multistep process, which includes endothelia cells proliferation, migration, vascular tubule formation, and cell survival [9]. During this process, VEGFA and ANGPT2 have been demonstrated to be the major regulators [11]. miR-218 has been reported to suppress tumor angiogenesis in gastric cancer [40]. While it is not clear if miR-218 maintains the same function in CRC. In our study, miR-218 was demonstrated to inhibit both protein and mRNA levels of VEGFA and ANGPT2. Moreover, when treated with supernatant from CRC cells after miR-218 transfection, the tube formation ability of HUVECs was significantly decreased. To our best knowledge, it is the first report that miR-218 acts as a suppressor for angiogenesis in CRC.

As miRNAs usually regulates multiple gens, it’s important to determine its targets in CRC. With the help of literature research and miRNA targets prediction database, CTGF was chosen as the candidate in our study. Previous studies have indicated that CTGF plays an important role in both EMT and angiogenesis processes. CTGF is a transcriptional target of TGF-β that interacts with other growth factors, extracellular matrix proteins, and cell surface proteins [18]. TGF-β works as a pro-metastatic role in CRC and is associated with poor outcomes [6]. A similar role has been proposed for CTGF for its correlation with worse prognosis [28]. TGF-β activates CTGF expression through canonical pSMAD2/3 pathway, which has been well studied for cancer development [26]. CTGF has also been reported to promote EMT process in Kawasaki disease by regulation of KLF4 [15]. In the process of angiogenesis, CTGF has been demonstrated to be the regulator of ANGPT2 expression, which is key factor for tumor angiogenesis [37]. Silencing CTGF expression significantly reduced tumor growth [7] and angiogenesis [37] in vivo. In this present study, CTGF was for the first time identified as the direct target of miR-218 by dual luciferase assay. Over expression of miR-218 in CRC cell lines could significantly inhibit both mRNA and protein levels of CTGF. To determine the role of CTGF in miR-218 induced regulation, we treated CRC cells with CTGF protein directly. Our data indicated that supplement of CTGF rescued the inhibition effects from miR-218 on EMT and angiogenesis proved by the reversed change in associated proteins. These data revealed that miR-218 inhibited EMT and angiogenesis processes in CRC cells through directly suppressing CTGF expression.

## Conclusion

CTGF has been widely studied and treatment of certain kinds of cancer by CTGF antibody is already under clinical trial [33]. Our results suggest that CTGF is a direct target of miR-218, which could suppress EMT and angiogenesis of CRC cells via directly inhibiting CTGF expression. These findings identify a new mechanism for miRNAs function in CRC progression and miR-218 might become a potential target for therapy in patients with CRC.
